# Efficacy and toxicity of CLAG combined with pegylated liposomal doxorubicin in the treatment of refractory or relapsed acute myeloid leukemia

**DOI:** 10.1002/cam4.5938

**Published:** 2023-05-10

**Authors:** Han Yao, Cheng Zhang, Xu Tan, Jieping Li, Xiaolin Yin, Xiaojuan Deng, Ting Chen, Jun Rao, Lei Gao, Peiyan Kong, Xi Zhang

**Affiliations:** ^1^ Medical Center of Hematology Xinqiao Hospital, Army Medical University Chongqing China; ^2^ State Key Laboratory of Trauma, Burns and Combined Injury Chongqing China; ^3^ Central Hospital of Changsha Changsha Hunan Province China; ^4^ The 303rd Hospital of the Chinese People's Liberation Army Nanning China

**Keywords:** cladribine, hematopoietic stem cell transplantation, pegylated liposomal adriamycin, refractory and relapsed AML, reinduction

## Abstract

**Background:**

Refractory and relapsed acute myeloid leukemia (r/rAML) is associated with a difficult prognosis; clinical trials are typically suggested despite lack of a recognized standard of care. Combinatorial chemotherapy regimens utilized for r/rAML salvage play a crucial role in battling this invasive phase. Although it is characterized by a low response rate, CLAG is a traditional regimen used in r/rAML. We aimed to compare the efficacy and toxicity of CLAG+PLD to explore whether there was any improvement with the addition of pegylated liposomal doxorubicin (PLD) to CLAG

**Methods:**

A total of 110 r/rAML patients were retrospectively analyzed from February 2017 to June 2020 at the Medical Center of Hematology, XinQiao Hospital, the 303rd Hospital of the Chinese People's Liberation Army, and Central Hospital of Chang Sha, Hunan Province. The response, overall survival (OS), disease‐free survival (DFS), and side effects in 110 r/rAML patients were evaluated retrospectively. Of these, 55 patients were administered CLAG+PLD, while 55 patients received CLAG alone as salvage therapy.

**Results:**

In the CLAG+PLD group, there were 27 (49.1%) cases of complete response (CR) with no measurable residual disease (MRD−), 12 (21.8%) cases of CR with positive MRD (MRD+), 5 (9.1%) cases of partial response (PR), 11 (20%) cases of no response (NR), and no cases of death during the cycles. The response rates in the CLAG group were lower: CR was reached in 24 (46.6%) patients with MRD−, 6 (10.9%) patients with MRD+, 10 (18.2%) patients with PR, 13 (23.6%) patients with NR, and 2 (3.6%) patients who passed away, one from infection and the other from cerebral hemorrhage. The median OS and DFS were not attained in the CLAG+PLD group during the 2‐year OS and DFS follow‐up, while both values were 10 months in the CLAG group (*p* = 0.023 and *p* = 0.045, respectively). The results of the Cox regression analysis for the CLAG+PLD group were strongly illustrative of the importance of hematopoietic stem cell transplantation (HSCT) following salvage therapy. No increased toxicity was observed in the CLAG+PLD group.

**Conclusion:**

CLAG+PLD is a potential salvage regimen for r/r AML that has a similar toxicity profile to CLAG and that improves response rates, 2‐year OS, and DFS relative to CLAG.

## 1 INTRODUCTION

Despite advancements in acute myeloid leukemia (AML) treatment, r/rAML still presents significant challenges. R/rAML has a poor prognosis, and treatment is difficult. Primary refractory or resistant AML refers to the 10% to 40% of newly diagnosed AML cases that do not achieve complete remission (CR) following rigorous induction treatment.^1^ In r/rAML, disease‐free survival (DFS) and overall survival (OS) will be suboptimal if patients do not achieve CR prior to HSCT or other consolidation therapy.^2^ Traditional cytotoxic chemotherapy, small molecules, niche‐targeting drugs, and an ever‐growing range of immunotherapeutic strategies are all choices for salvage therapy. Combinatorial chemotherapy regimens (both conventional and novel), which balance both efficacy and safety, are one of them and are crucial in halting this invasive phase.^3^

An analog named Cladribine is active against r/rAML. Pharmacological studies have demonstrated that cladribine before treatment promotes cytarabine intracellular absorption and cytarabine triphosphate (Ara‐CTP) buildup in AML blasts both in vitro and ex vivo.^4^ A traditional treatment for r/rAML, the CLAG regimen consists of cladribine and cytarabine with G‐CSF priming and has a CR rate of between 50% and 61.7%.^5^, ^6^, ^7^ Numerous investigations have shown that the addition of a different medication with a different mechanism can both increase efficacy and exert an equal amount of harm. According to a literature study, CLAG+X was more effective than CLAG alone in a variety of ways for both adults and children, and any side effects are tolerable. For the treatment of r/r AML, CLAG‐based regimens included CLAG+M (mitoxantrone), IAC (I, idarubicin), and C+CAG (aclacinomycin), among which the CR rate varied from 22% to 67.6%, and the total toxicity in most trials was acceptable (Table 1). Table 1 summarizes the Phase II and Phase III clinical trials concerning combinatorial chemotherapy using CLAG‐based regimens in the treatment of r/rAML that demonstrate promising efficacy.

**TABLE 1:** References on cladribine in the treatment of r/rAML.

Cohort	Study	Cases	Age (years, range)	Sex (F/M)	CR (%)	OS	DFS	Early death
CLAG+M	Wrzesien‐Kus et al.^7^	43	44 (20–66)	21/22	39.8	23.7 weeks	26.2 weeks	2 (5%)
	Wierzbowska et al.^8^	114	45 (20–66)	53/61	58	9 months	NA	8 (7%)
	Patzke et al.^9^	34	52 (20–67)	16/18	47.1	7 months	3	NA
	Halpern et al.^10^	60	61 (33–77)	25/35	60	NA	NA	NA
	Ruan et al.^11^	20	7 (1–14)	44,331	75	NA	NA	3 (15%)
	Scheckel et al.^12^	34	55.5 (21–73)	19/15	61.3	9.5 months	NA	0
CLAG	Bao et al.^6^	55	51 (33–69)	31/24	61.7	12.0 months	NA	NA
	Wrzesien‐Kus et al.^13^	58	45 (18–67)	30/28	50	34 weeks	17 weeks	NA
	Robak et al.^14^	20	44 (20–62)	10/10	50	24 weeks	22.5 weeks	NA
IAC	Wang et al.^15^	67	50.54 (32.8–58)	36/31	57.9	10 months	NA	NA
	Jain et al.^16^	46	55 (19–65)	NA	22	8.8 months	10.3 months	8 (15%)
	Fridle et al.^17^	34	51 (19–52)	38/23	52.9	NA	NA	2 (5.9%)
	Mayer et al.^18^	20	63 (43–80)	12/8	40	8.8 months	NA	1 (5%)
C+CAG	Wang et al.^5^	34	47 (18–72)	19/15	67.6	19 months	NA	NA

Abbreviations: C in CAG, aclacinomycin; C, cladribine; CR, complete response; I, idarubicin; M, mitoxantrone; NA, not available; OS, overall survival.

PLD, short for pegylated liposomal doxorubicin, exhibited a reduced cardiotoxicity and an improved pharmacokinetic profile compared to traditional doxorubicin applied in various malignancies.^19^ PLD was also associated with milder myeloid‐suppression and lower rates of infection compared to doxorubicin.

We have reported the application of CLAG+PLD in the successful treatment of a primary refractory blastic plasmacytoid dendritic cell tumors (BPDCN),^20^ and synergism anti‐leukemia effects were observed in vitro.^21^ Here, we aimed to compare the efficacy and toxicity of CLAG+PLD to explore whether there was any improvement with the addition of pegylated liposomal doxorubicin to CLAG.

## 2 METHODS

### 2.1 Study population

A total of 110 r/rAML patients were retrospectively analyzed from February 2017 to June 2020 at the Medical Center of Hematology, XinQiao Hospital, the 303rd Hospital of the Chinese People's Liberation Army, and Central Hospital of Chang Sha, Hunan Province. This was a retrospective matched case–control study involving two groups. We reviewed data and possible risk factors in these groups related to patient response in r/rAML. The controls in this matched case–control study were chosen based on factors that included the following: age, sex, leukocytes at diagnosis, ECOG, FAB classification, altered genes, risk stratification, and previous rounds of chemotherapy. CLAG+PLD was administered during the period of 2017 to June 2020. Patients in the control CLAG group were chosen retrospectively relative to the CLAG+PLD group according to the aforementioned matching rules. The follow‐up was 26 months (14.88–19.12), whereas, the follow‐up in the CLAG group was 16.2 months (13.39–19.01).

The CLAG+PLD group and the CLAG group each consisted of 55 patients, all of whom met the following inclusion criteria: (1) a diagnosis of AML based on morphology, immunology, cytogenetics, and molecular biology; (2) relapsed or refractory AML with more than 10% leukemia blasts and (3) receipt of CLAG+PLD or CLAG treatment according to disease conditions, clinical need, and the patients' willingness. If multiple lines of salvage therapy were performed after enrollment, only the first line was recorded and analyzed, which meant no patients were included repeatedly. Risk stratification was evaluated according to the 2017 ELN AML Guidelines based on patients' cytogenetics and molecular abnormalities.

The study protocol was approved by the Ethics Committee Review Board of the Second Hospital of Army Medical University. Since this was a retrospective analysis, a waiver/exemption regarding written informed was granted by the Ethics Committee.

### 2.2 Treatment

This is a retrospective study. The usual application dosages of PLD are 40 and 5 mg/m^2^ per week in multiple myeloma (MM).^22^, ^23^ However, the use of PLD in leukemia is scarcely reported. Given that PLD was being applied in combination with CLAG, which is an intensified regimen for r/rAML, the dose of PLD was tailored to 30 mg/m^2^ to minimize adverse effects. We propose to address the question of dose exploration in a subsequent prospective study.

The patients in the CLAG+PLD group received the following regimen: cladribine 5 mg/m^2^ and cytarabine 1–2 g/m^2^ as a continuous infusion over the course of 5 days (cytarabine can be tailored according to the score of the Eastern Cooperative Oncology Group (ECOG)); G‐CSF priming 5 μg/kg (days 1–5), omitted if the leukocyte count was above 20 × 10^9^/L; and PLD 30 mg/m^2^ as a continuous infusion. The CLAG group was treated with the classical CLAG regimen: cladribine 5 mg/m^2^/day (days 1–5); Ara‐C 2 g/m^2^/day (days 1–5); and G‐CSF priming 5 μg/kg (days 1–5), omitted if the leukocyte count was above 20 × 10^9^/L.

Target drugs such as BCL‐2 inhibitor and FLT3 inhibitor were chosen according to the gene features at the re‐induction; however, the target drug was tailored, then quickly stopped when myeloid‐supression occurred. To be more specific, the average duration of treatment with the target drug ranged from 5 to 12 days.

Consolidation after one cycle of CLAG+PLD or PLD was planned according to the patient's response and performance status. If HSCT was feasible and could be performed in a timely fashion, this was the preferred consolidation. If the patient was fit enough to receive a second cycle but HSCT was not planned or could not be scheduled until much later, a second cycle of chemotherapy was recommended.

### 2.3 Assessments and follow‐ups

CR was defined by the following criteria: bone marrow (BM) with at least 20% cellularity and under 5% BM blasts at steady state after chemotherapy, without cytological evidence of leukemia; no transfusion requirement; leukocyte count above 1 × 10^9^/L; platelet count above 100 × 10^9^/L; and lack of extramedullary disease. Partial remission (PR) was defined by a lack of extramedullary disease, along with any of the following: 5%–25% BM blasts, a decrease in BM blasts by at least 50%, or Auer rod positivity and <5% BM blasts. Aberrant leukemia‐associated immune phenotypes (LAIPs) detected by multiparameter flow cytometry (MFC) were primarily used to test for MRD in bone marrow samples; MRD‐positive status was defined as >0.01% of cells with a LAIP phenotype in bone marrow samples. The overall remission rate (ORR) was defined as the combined rate of CR and PR. Overall survival (OS) was calculated from the date of treatment to the time of death from any cause. DFS was defined as the time interval to the first event (relapse or death) and calculated from the date of treatment and the time of first event from any cause. The follow‐up duration was 24 months (range 0.5–45).

### 2.4 Next‐generation sequencing and analysis

The molecular abnormalities of all patients were analyzed before CLAG+PLD or CLAG salvage treatment. Genomic DNA was extracted using the EZNA Blood DNA Midi Kit (Omega Bio‐Tek). DNA samples were sequenced by next‐generation sequencing (NGS) using the MiSeq platform (Illumina); the target sequencing panel covered the entire coding sequences of 143 genes known to be relevant to AML pathogenesis, with an average effective depth ≥1000× in the target area.

### 2.5 Statistical analysis

The data cutoff for this report was January 2020. Assessments at screening served as baseline data. Descriptive statistics are presented as the mean ± standard deviation (mean ± SD) for continuous data and as numbers and percentages for dichotomous/categorical data. The chi‐square (*χ* ^2^) test or Fisher's exact test was used for categorical variables, and the Kruskal–Wallis test was used for continuous variables. Survival functions were estimated using the Cox regression method and Kaplan–Meier (KM) method and were compared using the log‐rank test. All analyses were performed using GraphPad Prism 9, SPSS 21.0 software (IBM, USA) and Office 2010 software (Microsoft, USA). A two‐sided *p* value <0.05 was considered statistically significant.

## 3 RESULTS

### 3.1 Baseline characteristics

The baseline characteristics of the patients are presented in Table 2. In total, 110 patients were analyzed in our study (each group consisted of 55 patients). In the CLAG+PLD group, the mean age was 34.56 (2–65) years; 24 patients were male, and 31 were female. Fourteen (25.5%) and 41 (74.5%) patients were diagnosed with relapsed and refractory AML, respectively; 4 (7%) among them were diagnosed with secondary AML (including chronic myeloid leukemia (CML), myeloid fibrosis (MF), and myeloproliferative neoplasm (MPN)). The use of TKIs was permitted in combined treatments. In the CLAG group, the median age was 30.57 (3.5–67) years; 19 patients were male, and 36 were female. Thirteen (23.6%) and 42 (76.4%) patients were diagnosed with relapsed and refractory AML, respectively; 5 (9%) among them had secondary AML (including 1 case of CML, 2 cases of MF, and 1 case of myelodysplastic syndrome (MDS)). Patients were classified as having favorable, intermediate, or adverse risks based on cytogenetics and molecular abnormalities as specified by the ELN risk standards. Table 3 shows the gene alterations classified as high risk for both groups. In both groups, most patients had an Eastern Cooperative Oncology Group (ECOG) performance status of 1 or 2. No significant difference was found in age, sex, leukocyte count, ECOG status, FAB type, number of altered genes, risk stratification, and cycles of previous treatments before treatment with CLAG+PLD or CLAG.

**TABLE 2:** Baseline characteristics of the CLAG+PLD group and the CLAG group.

Characteristic	CLAG+PLD	CLAG	
Age, years	34.56 (2–65)	30.57 (3.5–67)	*p* = 0.220
Sex, no. (%)			*p* > 0.999
F	31 (56.4%)	36 (64.5%)	
M	24 (43.6%)	19 (34.5%)	
Leukocyte count (1000/μL)	22.2 (1.09–730.0)	1.0 (0.85–282.8)	*p* = 0.058
ECOG PS, no. (%)			*p* = 0.480
0	0	0	
1	32	35	
2	20	19	
3	3	1	
4	0	0	
FAB classification, no. (%)			*p* = 0.216
M0	1	0	
M1	4	0	
M2	22	23	
M4	13	12	
M5	8	14	
M7	1	0	
MAL	5	1	
AML‐MRC	1	5	
Altered genes			*p* = 0.446
0	7	6	
1	17	13	
2	9	10	
3	5	13	
≥4	14	13	
Risk stratification			*p* = 0.613
Favorable	2	1	
Intermediate	19	17	
Adverse	34	35	
Previous treatment (cycles)			*p* = 0.391
2	26	33	
3	10	3	
4	7	8	
5	4	3	
≥6	8	8	

**TABLE 3:** High risk gene alteration between the groups.

Genetic alterations	CLAG+PLD	CLAG
p53 mutation	4 (7%)	3 (5%)
FLT3‐ITD	15 (27%)	17 (30%)
BCR/ABL	2 (4%)	0 (0)
KMT2A rearraged	2 (4%)	3 (5%)
DEK‐NUP214	3 (5%)	4 (7%)
GATA2/EVI1	3 (5%)	2 (4%)
Splicing mutation	13 (24%)	18 (33%)

### 3.2 Clinical efficacy of CLAG+PLD versus CLAG

**FIGURE 1:**
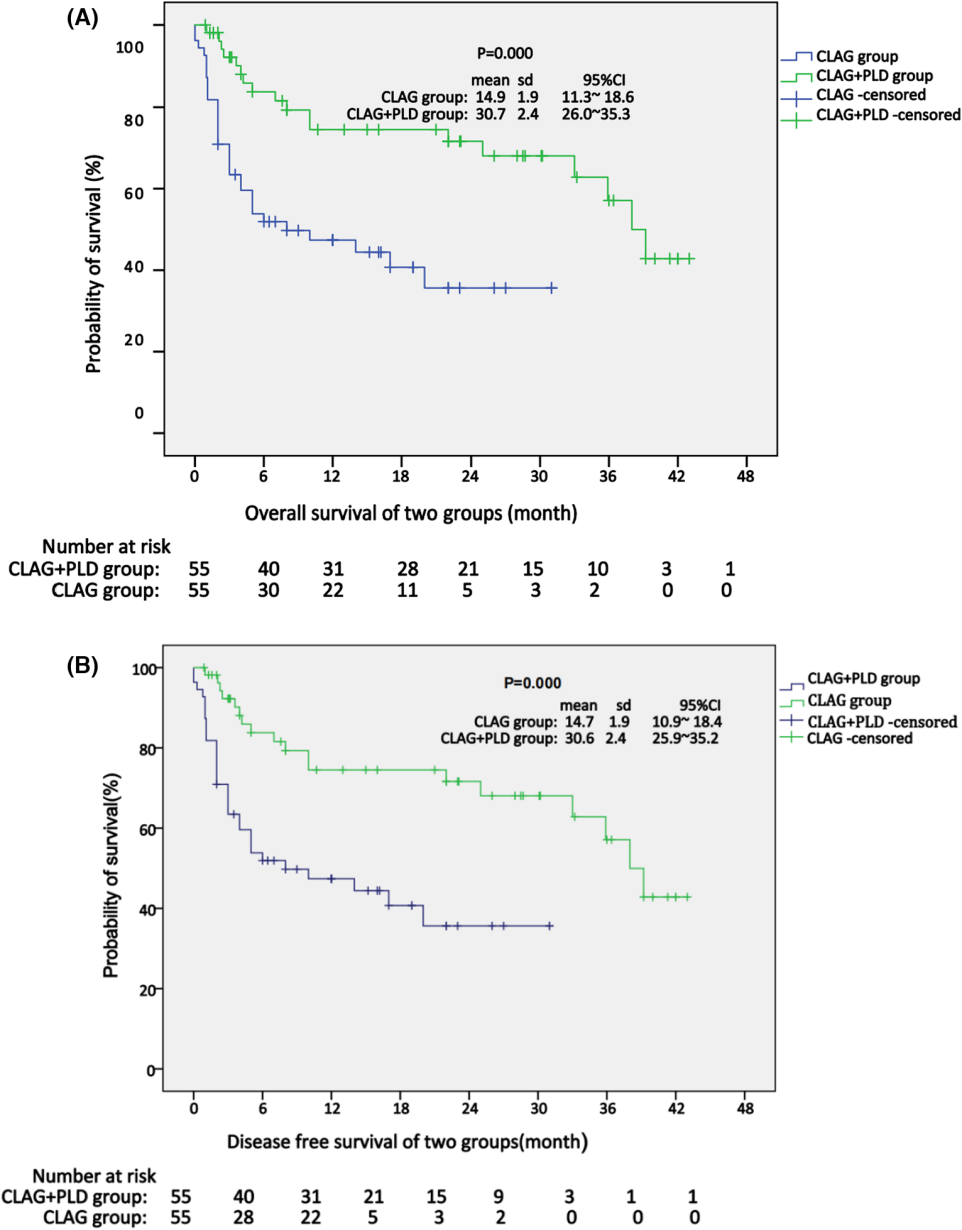
(A, B) Kaplan–Meier estimates of OS (months) and DFS (months) for all 110 r/rAML patients.

As shown in Table 4, in the CLAG+PLD group, 27 (49.1%) of patients achieved CR and were negative for measurable residual disease (MRD), 12 (21.8%) achieved CR but were positive for MRD, 5 (9.1%) achieved partial response, and 11 (20%) had no response. By contrast, in the *CLAG* group, CR with negative MRD, CR with positive MRD, partial response and no response were observed in 24 (46.6%), 6 (10.9%), 10 (18.2%), and 13 (23.6%) of patients, respectively (*p* = 0.007). Notably, 2 patients died in the CLAG group: one died of infection, and the other died of cerebral hemorrhage. Regarding overall survival (OS), the median survival was not reached in the CLAG+PLD group but was 10 months in the CLAG group, with a significance of *p* = 0.000. Similarly, the median disease‐free survival (DFS) was not reached in the CLAG+PLD group, while the value in the CLAG group was 10 months (*p* = 0.000) (Figure 1A,B).

**TABLE 4:** Clinical efficacy after salvage therapy with CLAG+PLD or CLAG by the response state of the patients after recovery of bone marrow.

Response to induction	CLAG+PLD	CLAG	*p* = 0.007
CR, MRD (−)	27 (49.1%)	24 (46.6%)	
CR, MRD (+)	12 (21.8%)	6 (10.9%)	
PR	5 (9.1%)	10 (18.2%)	
NR	11 (20%)	13 (23.6%)	
Death	0	2 (3.6%)	

Abbreviations: CR, complete response; MRD, minimal residue disease; NR, no response; PR, partial response.

### 3.3 Cox regression models of overall survival for the CLAG+PLD group

**FIGURE 2:**
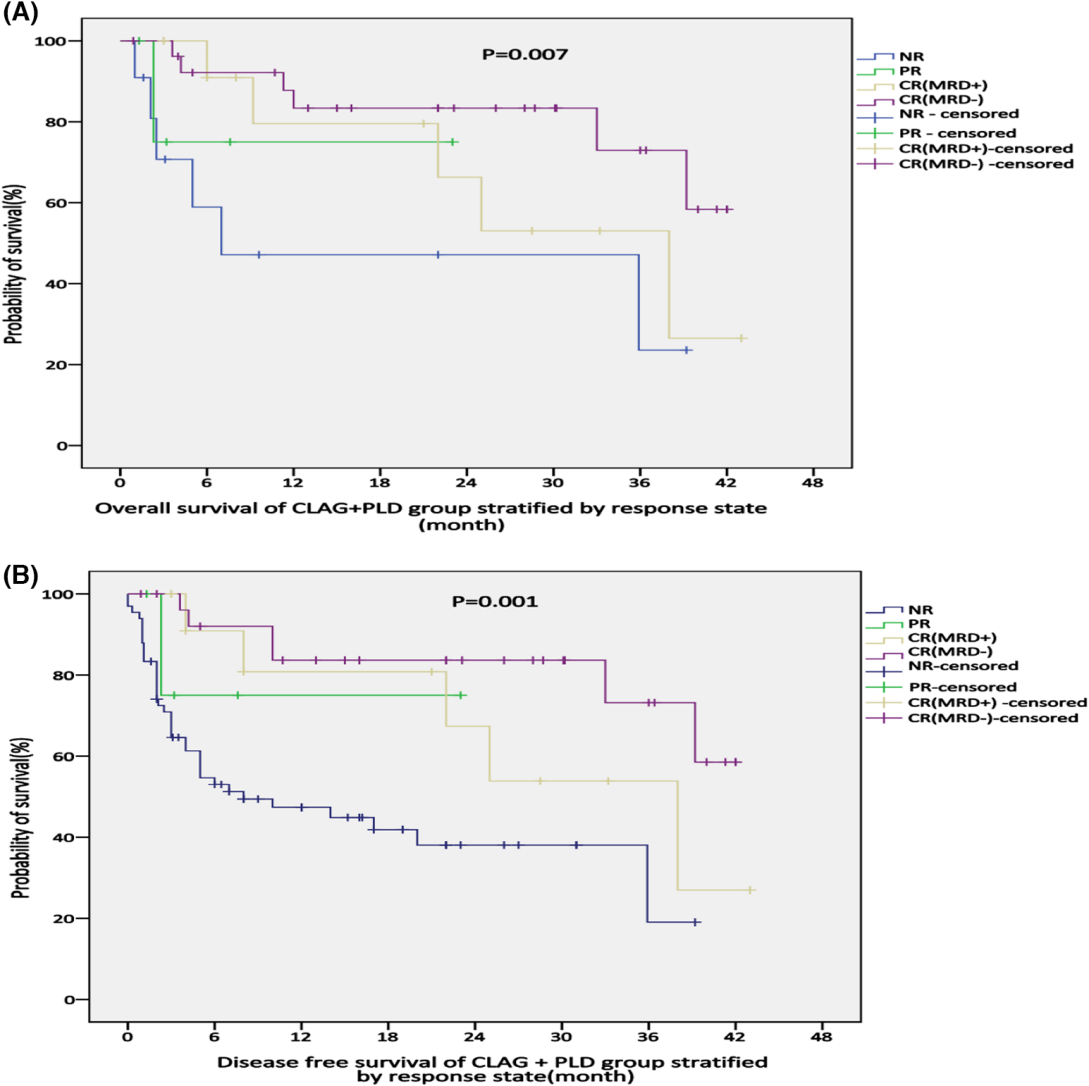
(A, B) Analysis of OS and DFS in the CLAG+PLD group according to the response state when treated with CLAG+PLD (*p* = 0.007, *p* = 0.001).

The CLAG+PLD group was further analyzed using Cox regression models; the covariates included leukocyte count, sex, cytogenetic risk stratification, previous cycles of treatment, response state when treated with CLAG+PLD, and whether HSCT was performed after salvage treatment (Table 5). The results showed that both HSCT and response state had a significant impact on OS, with *p* = 0.010 and 0.017, respectively. Furthermore, the KM survival curves of the CLAG+PLD group showed significant differences in OS and DFS when stratified by the variables of response state and whether patients underwent HSCT after salvage reinduction with CLAG+PLD (Figure 2A,B).

**TABLE 5:** Cox regression models of overall survival for the CLAG+PLD group.

Cox regression models on overall survival for group						
	B	SE	Wald	df	Sig.	Exp (B).
Age(years)	−0.865	0.468	3.424	1	0.064	0.421
Gender(female vs. male)	0.290	0.587	0.244	1	0.622	1.336
Leukocyte (1000/μL)	0.225	0.564	0.159	1	0.690	1.252
Cytogenetic risk	0.591	0.707	0.698	1	0.403	1.805
Cycles of previous chemotherapy	−0.315	0.685	0.211	1	0.646	0.730
Refractory or relapse	0.885	0.925	0.917	1	0.338	2.424
Transplantation or not	−1.647	0.637	6.683	1	0.010	0.193
Response state	−0.482	0.201	5.721	1	0.017	0.618

### 3.4 Clinical efficacy of CLAG+PLD stratified by HSCT

**FIGURE 3:**
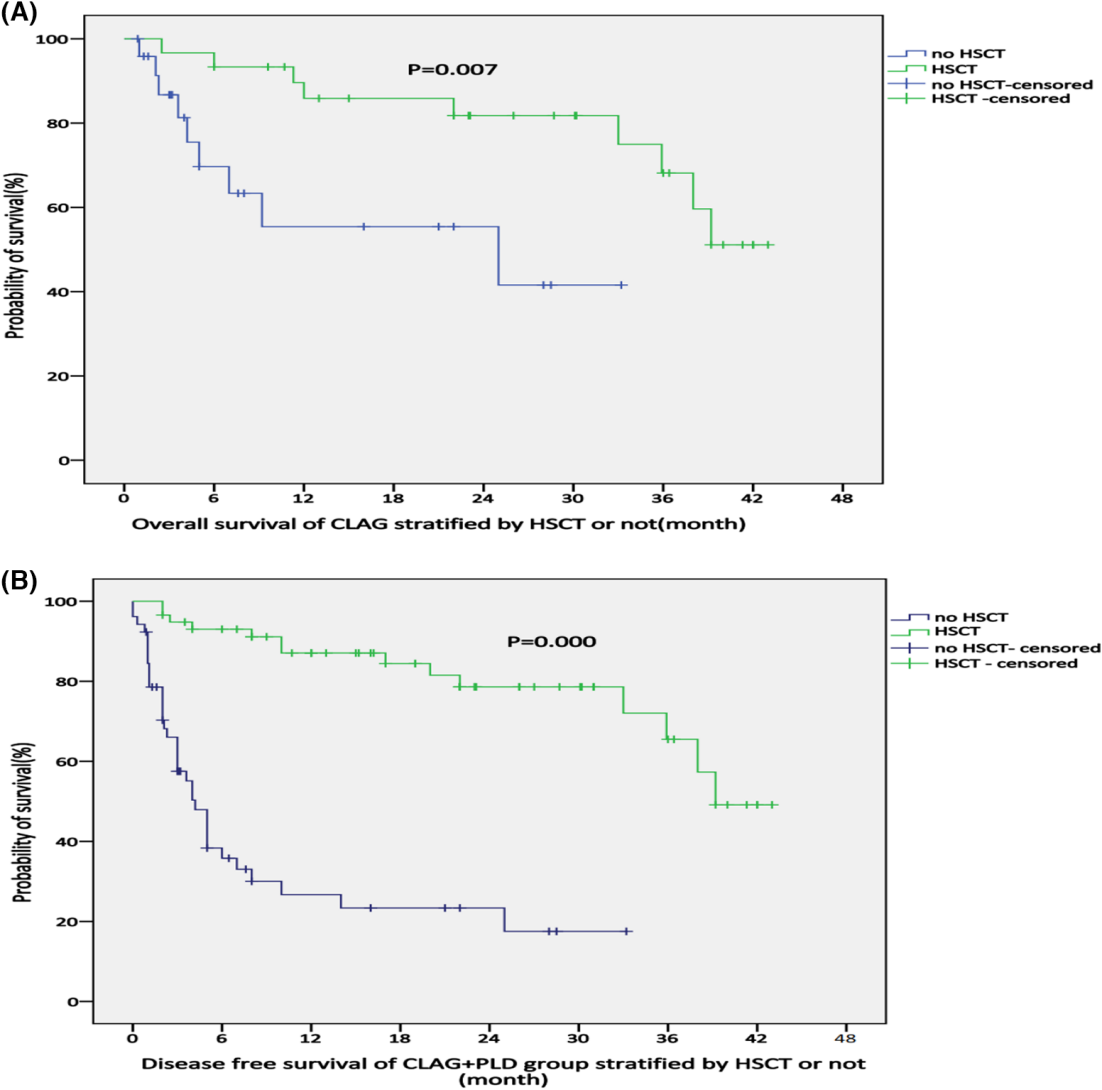
(A, B) OS and DFS of the CLAG+PLD group according to whether they received HSCT treatment (*p* = 0.007, 0.000).

**FIGURE 4:**
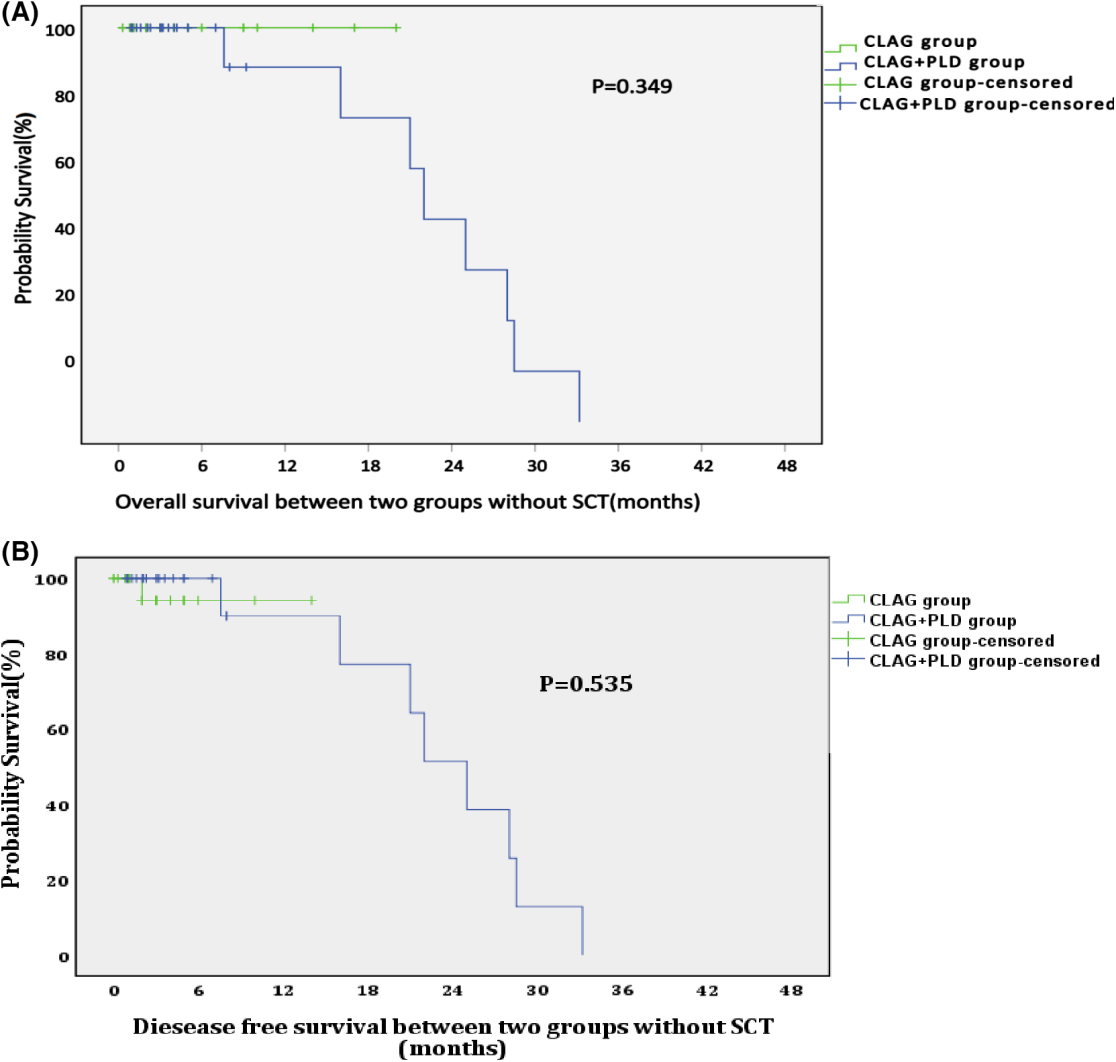
(A, B) OS and DFS of for both groups when censored at the time of SCT (*p* = 0.349, 0.535).

**FIGURE 5:**
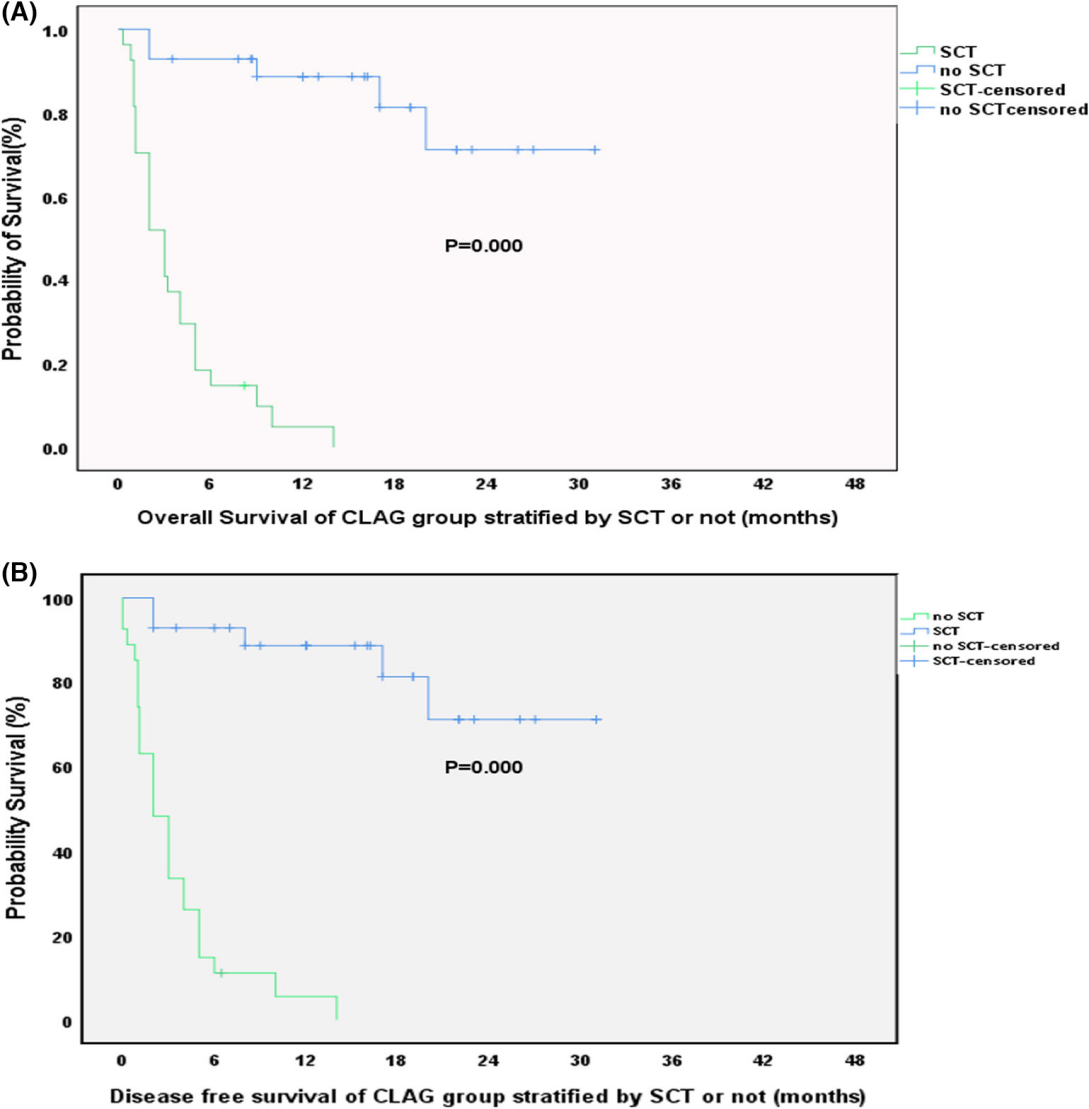
(A, B) OS and DFS of for CLAG group when stratified by SCT or not (*p* = 0.000, 0.000).

In the CLAG+PLD group, 30 patients underwent HSCT. Specifically, 11 patients received haplo‐HSCT, 6 received matched HSCT from unrelated donors, 12 received matched HSCT from related donors, and 1 received auto‐HSCT due to a lack of available donors. Twenty‐five patients did not receive HSCT. There was no significant difference among the above groups in terms of response upon treatment with CLAG+PLD (Table 5). However, both the OS and DFS in the HSCT group were superior to those in the non‐HSCT group at the 2‐year follow‐up (Figure 3). No significant difference in either OS or DFS was observed if patients in both groups were censored at the time of stem cell transplantation (SCT) (Figure 4A,B). In contrast, in the CLAG group, 28 patients underwent HSCT. Both the OS and DFS in the HSCT group were superior to those in the non‐HSCT group at the 2‐year follow‐up (Figure 5A,B).

### 3.5 Overall hematologic and nonhematologic toxicity

All patients experienced WHO grade 4 granulocytopenia, anemia, and thrombocytopenia. The CLAG+PLD and CLAG groups did not differ in terms of minimal neutrophil, platelet, or hemoglobin recovery. Severe (grades 3–4) infections occurred in 33% and 28% of the patients in the CLAG+PLD and CLAG groups, respectively (*p* = 0.56). Notably, 2 patients in the CLAG group died: one from infection and the other from cerebral hemorrhage. Other nonhematologic toxicities, including alopecia, infection, nausea/vomiting, sepsis, diarrhea, pneumonia, and asthenia, were infrequent and were not significantly different between the CLAG+PLD and CLAG groups. Details regarding hematologic and nonhematologic toxicity during induction treatment are provided in Table 6.

**TABLE 6:** Adverse events (AEs) among patients.

Adverse events	CLAG+PLD (55)	CLAG (55)	*p*
Hematologic			
Thrombocytopenia	55 (100%)	55 (100%)	>0.999
Febrile neutropenia	42 (76%)	40 (73%)	0.17
Neutropenia	55 (100%)	55 (100%)	>0.999
Anemia	55 (100%)	55 (100%)	>0.999
Nonhematologic			
Alopecia	12 (22%)	10 (18%)	0.77
Infection	18 (33%)	15 (28%)	0.56
Nausea/vomiting	26 (47%)	28 (51%)	0.11
Sepsis	3 (5%)	4 (7%)	0.29
Diarrhea	3 (3%)	0 (0%)	0.16
Pneumonia	5 (10%)	7 (13%)	0.87
Asthenia	14 (26%)	10 (19%)	0.43

## 4 DISCUSSION

Described in Table 1 are several clinical trials that involved the attempted use of different combinations of CLAG plus X to improve the response rate of patients with r/rAML. By searching PubMed, Embase, Web of Science, and Scopus, we found that none of the trials involved CLAG+PLD, and, yet, there is still much room for improvement in r/rAML treatment. We aimed to compare the efficacy and toxicity of CLAG+PLD and determine whether there was any improvement in treatment when pegylated liposomal doxorubicin was added to CLAG.

In this research, treatment with CLAG+PLD produced a higher CR rate and longer OS and DFS than CLAG (Table 4, Figures 1 and 2). In our research, the total CR rate in the CLAG+PLD group was 70.9%, which is comparable to previously reported CLAG+M and C+CAG rates of 75% and 67.6%, respectively, and there was no increased toxicity. Additionally, larger studies are expected to validate these findings. The ongoing prospective phase III clinical trial (ChiCTR1800017569) is still recruiting patients. Moreover, targeted drug like BCL‐2 inhibitor or FLT3 inhibitor were chosen according to the gene feature at the re‐induction, however, the targeted drug was tailored, then quickly stopped when myeloid‐supression occured. The difference brought on by the targeted medicine was thought to be minimal because the total duration was short.

Consistently positive MRD after induction treatment is associated with a poor prognosis in acute lymphoblastic leukemia (ALL)^24^; however, positive MRD in AML has not been addressed in the guidelines.^25^, ^26^ Several studies have found a higher relapse rate in MRD‐positive than MRD‐negative AML patients.^27^, ^28^, ^29^ Our retrospective analysis also confirmed that, for r/rAML, the MRD‐positive group had a worse prognosis than the MRD‐negative group (Figure 2). In our research, a higher proportion of MRD negativity was observed in the CLAG+PLD group than in the CLAG group (49.1% vs. 46.6, *p* = 0.007), indicating a better depth of remission.

Through Cox regression analysis, we found that outcomes did not significantly differ between relapsed AML and refractory AML patients or according to the number of previous treatment cycles, risk stratification, leukocyte count at diagnosis, or sex (Table 5); furthermore, it appears that a following HSCT and the response state are the two main factors affecting the DFS of patients treated with CLAG+PLD (*p* = 0.10 and 0.17). It is obvious that a better response is related to OS of r/rAML. HSCT still plays a vital role in the survival of patients with r/rAML. Although there was no significant difference in response status before HSCT in the CLAG+PLD group, HSCT extended both OS and DFS (Figure 3). In contrast, for the CLAG group, a superiority of OS and DFS was also observed in patients who underwent HSCT (both *p* < 0.000) (Figure 5). Therefore, reaching CR after either CLAG+PLD or CLAG requires prompt HSCT. Nevertheless, improvements in HSCT are urgently needed; for example, the optimal timing, conditioning regimen, and donor choice (when there is more than one available donor) need to be identified.^8^, ^9^

The overall toxic side effects of CLAG+PLD were not significantly greater than those of CLAG (Table 6). Patients may benefit from a tailored dose of cytarabine; specifically, in our application of CLAG+PLD, cytarabine was tailored to 1 g/m^2^, not 2 g/m^2^ as in classical CLAG. Another aspect is that PLD exhibits reduced cardiotoxicity and an improved pharmacokinetic profile with milder myeloid suppression and a lower rate of infections than doxorubicin. The CLAG+PLD regimen may be safer for patients with heart dysfunction due to its milder cardiotoxicity.

This was a retrospective clinical study, with all the inherent limitations of such a design. First, the number of included patients was limited. Second, since a wide variety of agents are currently available; improving the response rate by combining our CLAG+PLD regimen with novel agents may be a potential treatment strategy for r/rAML. Thus, additional work is in progress to dissect the underlying biology in preclinical models and multicenter clinical trials.

## AUTHOR CONTRIBUTIONS

**Han Yao:** Conceptualization (equal); data curation (lead); project administration (equal); writing – original draft (lead). **Cheng Zhang:** Conceptualization (equal). **Xu Tan:** Data curation (equal); formal analysis (equal); investigation (equal). **Jieping Li:** Data curation (equal); project administration (equal). **Xiaolin Yin:** Methodology (equal). **Xiaojuan Deng:** Methodology (equal). **Ting Chen:** Data curation (equal); formal analysis (equal). **Jun Rao:** Investigation (equal); methodology (equal). **Lei Gao:** Project administration (equal); supervision (equal). **Peiyang Kong:** Conceptualization (lead); project administration (equal). **Xi Zhang:** Conceptualization (equal); funding acquisition (equal); investigation (equal).

## Data Availability

The data that support the findings of this study are available from the corresponding Xi Zhang, upon reasonable request.
